# The role of Schlafen 11 (SLFN11) as a predictive biomarker for targeting the DNA damage response

**DOI:** 10.1038/s41416-020-01202-y

**Published:** 2020-12-16

**Authors:** Niamh Coleman, Bingnan Zhang, Lauren A. Byers, Timothy A. Yap

**Affiliations:** 1 Department for Investigational Cancer Therapeutics (Phase I Program), University of Texas MD Anderson Cancer Center, Houston, TX USA; 2 Division of Cancer Medicine, University of Texas MD Anderson Cancer Center, Houston, TX USA; 3 University of Texas MD Anderson Cancer Center, Houston, TX USA; 4 Department of Thoracic/Head and Neck Medical Oncology, University of Texas MD Anderson Cancer Center, Houston, TX USA; 5 Institute for Applied Cancer Science, University of Texas MD Anderson Cancer Center, Houston, TX USA; 6 Khalifa Institute for Personalized Cancer Therapy, University of Texas MD Anderson Cancer Center, Houston, TX USA

**Keywords:** Cancer, Molecular biology

## Abstract

The therapeutic landscape of drugs targeting the DNA damage response (DDR) is rapidly expanding; however, an urgent unmet need remains for validated predictive biomarkers of response. SLFN11 has emerged as a promising predictor of sensitivity to DNA-damaging chemotherapies, and recently, been associated with sensitivity to PARP inhibition. We discuss its use as a predictive biomarker of response for targeting the DDR.

## Main

The therapeutic landscape of drugs targeting the DNA damage response (DDR) is rapidly expanding, yet there remains an urgent unmet clinical need for analytically validated predictive biomarkers of response beyond *BRCA1* and *BRCA2* mutations and a better understanding of resistance mechanisms for optimal patient selection and management.

Schlafen 11 (SLFN11), a putative DNA/RNA helicase that is recruited to the stressed replication fork and irreversibly triggers replication block and cell death, has emerged as a promising predictor of sensitivity to cytotoxic chemotherapies, specifically DNA-damaging agents (DDA), such as topoisomerase (TOP) I and TOP II (irinotecan and etoposide, respectively), DNA synthesis inhibitors (e.g. gemcitabine) and DNA cross-linkers and alkylating agents (e.g. cisplatin).^1,2^ Most recently, SLFN11 has also been associated with sensitivity to poly(ADP-ribose) polymerase (PARP) inhibitors.^3–7^

**Fig. 1:**
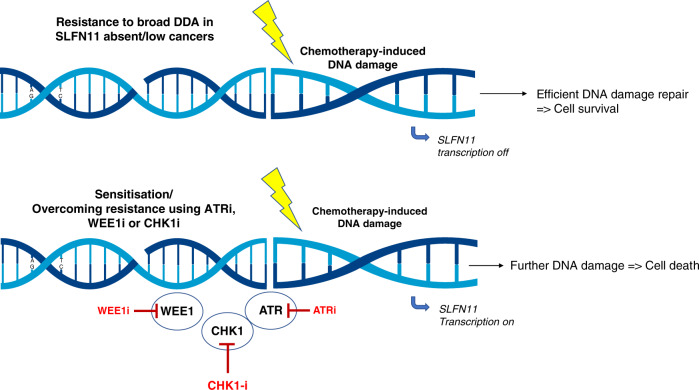
Summary of the resensitisation strategy of Winkler and co-workers. In SLFN11-low cancers, DDA combinations with DDR inhibitors, such as ATR, WEE1 or CHK1 inhibitors, could reverse resistance to broad DDA by targeting the replication stress response, inducing further DNA damage and ultimately leading to cell death.

The study by Winkler and co-workers^8^ in this issue of the *British Journal of Cancer* that accompanies this Editorial is both important and timely. Using an orthogonal multidisciplinary approach combining analyses of different cancer types using multiple models, combination strategies and mechanistic studies, it reinforces previously published work, while providing promising preclinical data supporting the use of SLFN11 as a predictive biomarker of DDA response (Fig. 1). Beyond this, it also offers potential novel treatment combinations of DDA with selected inhibitors against the DDR to overcome resistance.

Overall, their data are clear: (1) SLFN11 correlates with the response to different DDA, with the correlation significantly lower for some DDR inhibitors and absent with non-DNA-damaging anticancer drugs, and (2) novel drug sensitisation strategies for SLFN11-low cancers include DDA combinations (specifically gemcitabine) with some DDR inhibitors, such as ATR, WEE1 or CHK1 inhibitors, but not inhibitors of other key components along the DDR pathway (ATM, DNA-PK and PARP). Interestingly, two recent clinical trials reported that ATR or WEE1 inhibitor combinations with gemcitabine are potentially efficacious in ovarian and pancreatic cancer.^9,10^ Winkler and co-authors^8^ show in their preclinical models that these two tumour types present low or absent SLFN11 expression, highlighting the potential importance of SLFN11 in these cancer subtypes.

The authors also noted two surprising observations: (1) SLFN11 protein levels did not decrease following chemotherapy treatment, unlike the previous observations,^5,11^ and (2) PARP inhibitors, specifically olaparib, had a limited impact on SLFN11 in the models tested, contradicting other published data.^4,6,7^ Therefore, while promising, it is clear that the prospective clinical qualification of SLFN11 in tumour-specific settings is urgently required to confirm if it is a bona fide predictive biomarker, and to truly ascertain if the efficacy of such combination regimens may indeed be attributed to low or absent tumour SLFN11.

There are notable ongoing efforts to clinically validate SLFN11 in small-cell lung cancer (SCLC). In several preclinical studies using cell lines and patient-derived xenograft models, SLFN11 expression strongly predicted cisplatin and PARP inhibitor responses.^4,5,11^ In a Phase 2 trial of temozolomide plus veliparib versus temozolomide/placebo in patients with relapsed SCLC, SLFN11 expression was detected in approximately 50% of tumours using immunohistochemistry (IHC). In the temozolomide plus veliparib arm, the SLFN11-positive cohort had significantly prolonged progression-free survival (PFS) and overall survival (OS) compared with the SLFN11-negative group. However, SLFN11 was not associated with a difference in outcomes in patients treated on the temozolomide plus placebo arm, consistent with preclinical data showing that SLFN11 expression in cell lines does not predict for response to temozolomide.^7^ This implies that SLFN11 is a potential predictive biomarker for PARP inhibitor benefit in patients with SCLC. However, prospective validation of SLFN11 is still needed to confirm its biomarker status.

In the recently initiated Phase 2 randomised trial assessing maintenance atezolizumab in combination with talazoparib versus atezolizumab alone in patients with SLFN11-positive extensive-stage SCLC (ES-SCLC) (SWOG1929, NCT04334941), all patients will receive standard front-line induction therapy with platinum–etoposide plus atezolizumab, and prospectively screened for SLFN11 positivity. If SLFN11 expression is positive by IHC, patients will be eligible to enter the trial in the atezolizumab maintenance phase and be randomised to one of two arms, with or without talazoparib. The primary objective is to compare the progression-free survival between patients in both arms, with secondary objectives of overall survival, objective response and frequency of adverse effects. As also noted by the authors of this paper^8^, assessing SLFN11 expression by IHC is clinically feasible because it can be easily assessed as positive (H score > 1) or negative, and has been found positive in ~50% of ES-SCLC, as demonstrated in a previous trial.^7^ This will be the first trial to assess SLFN11 prospectively as a biomarker to select patients; therefore, the outcome will elucidate if patients with SLFN11-positive status derive additional benefit from PARP inhibitors in ES-SCLC. Of note, the authors of this paper and others have observed potentially higher in vitro correlation of SLFN11 with ‘PARP-trapping’ PARP inhibitors, which may effectively make them function as DDAs. Therefore, the predictive value of SLFN11 may vary among the different clinically available PARP inhibitors, depending on their degree of PARP trapping, with talazoparib the PARP inhibitor with the greatest PARP-trapping capacity observed preclinically.^12^

In addition to SCLC, SLFN11 has also shown promise as a predictive biomarker of response in ovarian and prostate cancer.^2,13^ Nogales and colleagues demonstrated that patients with ovarian and non-SCLC with SLFN11 hypermethylation had a poor response to both cisplatin and carboplatin.^2^ The overexpression of SLFN11 has also recently been shown to be a promising predictive biomarker of response in patients with castration-resistant prostate cancer (CRPC) to DDAs, including platinum-based chemotherapy.^13^ In this retrospective study, patients with SLFN11-positive CRPC had improved radiographical PFS and prostate-specific antigen (PSA) tumour marker responses compared with patients without SLFN11 overexpression, regardless of the presence or absence of DNA repair gene alterations and tumour histology (i.e. adenocarcinoma or neuroendocrine CRPC).^10^

Clinically, the use of SLFN11 protein expression by IHC as a predictive biomarker may present technical challenges, such as inherent intra- and inter-tumour heterogeneity, the requirement of fresh and contemporaneous tumour biopsy for real-time assessment. Previous studies assessing homologous recombination repair protein expression were limited by small numbers or technical issues, with poor reproducibility.^14^

Looking into the future, both gene and protein analyses as a DDA biomarker will require further rigorous research and clinical validation in order to optimise the efficacy of DDAs. We eagerly await the prospective clinical validation of SLFN11 as a bona fide predictive biomarker of response for optimal patient selection in SCLC and beyond.

## Data Availability

Not applicable.

## References

[1] Zoppoli G, Regairaz M, Leo E, Reinhold WC, Varma S, Ballestrero A (2012). Putative DNA/RNA helicase Schlafen-11 (SLFN11) sensitizes cancer cells to DNA-damaging agents. Proc. Natl Acad. Sci. USA.

[2] Nogales V, Reinhold WC, Varma S, Martinez-Cardus A, Moutinho C, Moran S (2016). Epigenetic inactivation of the putative DNA/RNA helicase SLFN11 in human cancer confers resistance to platinum drugs. Oncotarget.

[3] Murai J, Feng Y, Yu GK, Ru Y, Tang SW, Shen Y (2016). Resistance to PARP inhibitors by SLFN11 inactivation can be overcome by ATR inhibition. Oncotarget.

[4] Lok BH, Gardner EE, Schneeberger VE, Ni A, Desmeules P, Rekhtman N (2017). PARP Inhibitor activity correlates with slfn11 expression and demonstrates synergy with temozolomide in small cell lung cancer. Clin. Cancer Res..

[5] Stewart CA, Tong P, Cardnell RJ, Sen T, Li L, Gay CM (2017). Dynamic variations in epithelial-to-mesenchymal transition (EMT), ATM, and SLFN11 govern response to PARP inhibitors and cisplatin in small cell lung cancer. Oncotarget.

[6] van Erp AEM, van Houdt L, Hillebrandt-Roeffen MHS, van Bree NFHN, Flucke UE, Mentzel T (2020). Olaparib and temozolomide in desmoplastic small round cell tumors: a promising combination in vitro and in vivo. J. Cancer Res Clin. Oncol..

[7] Pietanza MC, Waqar SN, Krug LM, Dowlati A, Hann CL, Chiappori A (2018). Randomized, double-blind, phase II study of temozolomide in combination with either veliparib or placebo in patients with relapsed-sensitive or refractory small-cell lung cancer. J. Clin. Oncol..

[8] Winkler, C., Armenia, J., Jones, G. N., Tobalina, L., Sale, M. J., Petreu, T. et al. SLFN11 informs on standard of care and novel treatments in a wide range of cancer models. Br J Cancer 10.1038/s41416-020-01199-4 (2020). 10.1038/s41416-020-01199-4 PMC7921667 33339894

[9] Cuneo KC, Morgan MA, Sahai V, Schipper MJ, Parsels LA, Parsels JD (2019). Dose escalation trial of the WEE1 inhibitor adavosertib (AZD1775) in combination with gemcitabine and radiation for patients with locally advanced pancreatic cancer. J. Clin. Oncol..

[10] Konstantinopoulos PA, Cheng SC, Wahner Hendrickson AE, Penson RT, Schumer ST, Doyle LA (2020). Berzosertib plus gemcitabine versus gemcitabine alone in platinum-resistant high-grade serous ovarian cancer: a multicentre, open-label, randomised, phase 2 trial. Lancet Oncol..

[11] Gardner EE, Lok BH, Schneeberger VE, Desmeules P, Miles LA, Arnold PK (2017). Chemosensitive relapse in small cell lung cancer proceeds through an EZH2-SLFN11 axis. Cancer Cell.

[12] Murai J, Huang SYN, Das BB, Renaud A, Zhang Y, Doroshow JH (2012). Trapping of PARP1 and PARP2 by clinical PARP inhibitors. Cancer Res..

[13] Conteduca V, Ku SY, Puca L, Slade M, Fernandez L, Hess J (2020). SLFN11 Expression in advanced prostate cancer and response to platinum-based chemotherapy. Mol. Cancer Ther..

[14] Vollebergh MA, Jonkers J, Linn SC (2012). Genomic instability in breast and ovarian cancers: Translation into clinical predictive biomarkers. Cell. Mol. Life Sci..

